# Prevalence of Mandibular Third Molar Impaction, Associated Pathologies, and Correlation With Temporomandibular Joint Morphology in a Hospital‐Based Spanish Cohort: A Panoramic Radiography Study

**DOI:** 10.1155/ijod/6940859

**Published:** 2026-01-19

**Authors:** Hassan Ahmed Assiri, Albert Estrugo-Devesa, Sonia Egido-Moreno, Xavier Roselló Llabrés, Mohammad Shahul Hameed, Abdullah Alqarni, Jose López-López

**Affiliations:** ^1^ Department of Diagnostic Sciences and Oral Biology and Periodontology, College of Dentistry, King Khalid University, Abha, 61421, Saudi Arabia, kku.edu.sa; ^2^ Department of Odontostomatology, Faculty of Medicine and Health Sciences, School of Dentistry, University Campus of Bellvitge, University of Barcelona, Barcelona Dental Hospital [HOUB], Barcelona, 08970, Spain, ub.edu

**Keywords:** condyle, impaction, mandible, panoramic radiograph, temporomandibular joint, third molar

## Abstract

**Background:**

Mandibular third molar is the most frequent impacted tooth in the oral cavity. Its presence can be associated with complications including the temporomandibular joint (TMJ) symptoms. Therefore, the present study aimed to assess the prevalence of impacted mandibular third molar (IMTM), associated pathologies, and its correlation with TMJ morphology in a hospital‐based Spanish cohort.

**Methods:**

We retrospectively reviewed existing orthopantomographs (OPGs) records, panoramic images of patients aged ≥18 with at least one IMTM who attended the Dental Hospital of the University of Barcelona (HOUB) between September 2021 and May 2023. The OPGs were assessed and interpreted by an experienced oral and maxillofacial radiologist for the type of impaction according to Winter’s classification system, associated pathologies, and shape of mandibular condyle.

**Results:**

Out of 80 OPGs, 60% (95% confidence interval [CI]: 48.4%−70.7%) were females, and the majority 53.8% (95% CI: 42.3–64.9) were between 18 and 28 years of age. The prevalence rate of IMTM was 86.88%, with the left side commonly involved. On both sides, oval‐shaped condyle and vertical IMTM were the most common, with dental caries and bone loss being the frequently observed pathologies. Sclerotic changes were depicted in 15% (95% CI: 8.2%−24.7%) of the cases on both sides of TMJ. On the other hand, no statistically significant associations were noticed between the pathologies and condyle shape (*p* > 0.05, Cramér’s *V* < 0.25). Vertical and mesioangular, followed by horizontally IMTMs, were the most prevalent types of impactions, indicating nonsignificant association with condylar shape (*p* > 0.05, Cramér’s *V* = 0.21–0.23).

**Conclusion:**

In this hospital‐based cohort, vertical IMTM and oval condylar morphology were predominant; however, condylar shape did not correlate with impaction type on panoramic radiographs. The findings are preliminary and require validation in sufficiently powered cone beam computed tomography (CBCT)‐based studies with clinical TMJ assessment.

## 1. Background

Teeth play an important role in maintaining the health of the oral cavity and the general well‐being of an individual. When teeth fail to erupt and achieve their anatomical position and physiological functions, they are regarded as impacted teeth. According to the World Health Organization (WHO), an impacted tooth is defined as a tooth that cannot erupt in its normal occlusion because of hindrances in its eruption path in relation to factors such as overlying soft tissue, bone blocking the pathway, or any preexisting supernumerary or ankylosed [1]. The commonly named (wisdom tooth) or third molar is the only tooth that erupts during the adolescent stage or sometimes in adulthood. The timings of third molar development are diverse, providing that the calcification takes place at the age of 5 years, and the radiographic appearance is noticed by the 8–9 years of age [2]. In this regard, third molar tooth is considered the most frequently impacted tooth with reported prevalence rate of 16.7–68.6% [3]. Different theories explained the fact behind the phenomena of third molars impaction, including Mendelian, phylogenetic, and orthodontic theories [4]. The prevalence and pattern of impacted mandibular third molar (IMTM) varied with different geographical area and population [5]. In accordance with gender predilection, a study conducted by Chu et al. [6] reported female propensity. IMTM can be unilateral or bilateral. According to Winter’s classification, the pattern of the impacted third molar is determined by the angle formed between the intersecting longitudinal axis of the third and second molars. Therefore, distoangular impaction ranged between −11° and −79°, mesioangular impaction ranged between 11° and 79°, horizontal impaction ranged between 80° and 100°, and vertical impaction is ranged between 10° and −10° [1, 7]. Other rare positioning of the mandibular third molar impaction such as inverted and transversal orientations has been documented in the literature [8].

The conventional two‐dimensional imaging tools including intraoral periapical radiographs and extraoral orthopantomographs (OPG) remain the routinely diagnostic methods for the visualization of the IMTMs [9]. OPG displays the angulation, location, and relation of impacted molars to the adjacent structures [10]. It has been found that impacted third molars can induce various pathologies or remain asymptomatic in the oral cavity [11]. Accordingly, the most frequent third molar‐associated pathologies are dental caries, pericoronitis, periodontal issues, odontogenic cysts, root resorption, and tumors [12]. Thus, these pathologies are influenced by the impacted tooth’s position, eruption status, and angulation [13]. To our knowledge, the literature lacks studies that correlate the type of impaction with different associated pathologies and the impact on the shape of the mandibular condyle. Thus, the present study aimed to assess the prevalence of IMTMs, associated pathologies, and their correlation with temporomandibular joint (TMJ) morphology in the Spanish population. This study was conducted to establish more relevant information regarding the influence of impacted teeth on the shape of the condyle. We hypothesize that the different types of impactions are associated with different pathologies and have an impact on the condyle shapes.

Although studies have documented condylar various shapes on panoramic OPGs; the association between these morphological variations and the pattern of mandibular third molar impaction remains ambiguous and generally unexplored [14–16]. Consequently, we performed an exploratory analysis of IMTM patterns and associated pathologies in addition to investigating the correlation between condylar morphology impaction type in adult patients at a Spanish university dental facility.

## 2. Methods

### 2.1. The Aim, Design, and Setting of the Study

The study aim is to explore the prevalence of IMTMs, associated pathologies, and their correlation with TMJ morphology in the Spanish population. This retrospective study was conducted on the existing record of patient’s OPGs of visiting the dental Hospital of the University of Barcelona (HOUB) who aged ≥18 years and reported between September 2021 and May 2023. The protocol was approved by the institutional ethical committee of HOUB (protocol number: 2022‐032‐1). The study is conducted in accordance with the principles of the Declaration of Helsinki.

### 2.2. Characteristic of Participants

The patient’s consent form is waived as this is an anonymous retrospective analysis of the images. Data were collected from the patients subjected to the required panoramic radiographs (for various diagnostic purposes) done in the Radiology division of Dental College in Barcelona, Spain, using simple random sampling. OPG scans were performed as advised by their concerned doctors. The patient’s identity was protected using a codification system by one coinvestigator. We randomly selected 500 OPG from the HOUB archive, covering the period from September 2021 to May 2023. The inclusion criteria consisted of individuals aged 18 years or older, possessing diagnostic image quality and having at least one IMTM. Exclusion criteria included previous mandibular condylar trauma or surgery, significant deformity or artifact obstructing condyle visualization, or the absence of IMTM. Eighty radiographs fulfilled the criteria and were examined. A simple random sampling is followed to retrieve the 500 OPGs. Among them, 80 images met the inclusion criteria and were subjected to the study’s analysis. The patients were divided into four age groups: 18–28, 29−39, 40–50, and > 50 years old.

### 2.3. Image Acquisition

The OPG images were obtained using a Planmeca ProMax X‐ray unit (Planmeca Oy, 00880 Helsinki, Finland), equipped with a digital sensor called Planmeca Dimax 3. Images were acquired based on the manufacturer’s recommendations at 64–70 kV and 7–14 mA, depending on the patient’s gender and age. The recommended settings varied for adult females, small adults, and large adults.

### 2.4. Measurement Methods

Well‐trained doctoral students from the oral radiology department conducted the analysis of the condyle shapes, type of impaction, and impaction associated pathology twice, with a 2‐month interval between each session. The intra‐examiner reliability was calculated using the kappa statistic. Kappa results indicated a substantial agreement (*k* = 0.68). The radiographs were screened for the shape of mandibular condyle (oval, bifid, flattened, diamond, mixed, and crooked finger types) on both the right and left sides of the mandible as described by Gupta et al. [14]. In addition, the pathological changes including dental caries, bone loss distal to the second molar of more than 3 mm measured from the cementoenamel junction to the alveolar crest, external resorption of the second molar roots and neoplastic changes, were determined and correlated with the impaction type and the different shapes of the condyle. Cropped images of OPGs revealing different shapes of condyle are presented in Figure 1. The different Winter’s classification of IMTM is illustrated in Figure 2.

Figure 1Panoramic sections of different condyle shapes. (A) Flat shape. (B) Diamond shape. (C) Bifid shape. (D) Mixed shape. (E) Oval shape. (F) Crooked‐finger shape.

**Figure:**
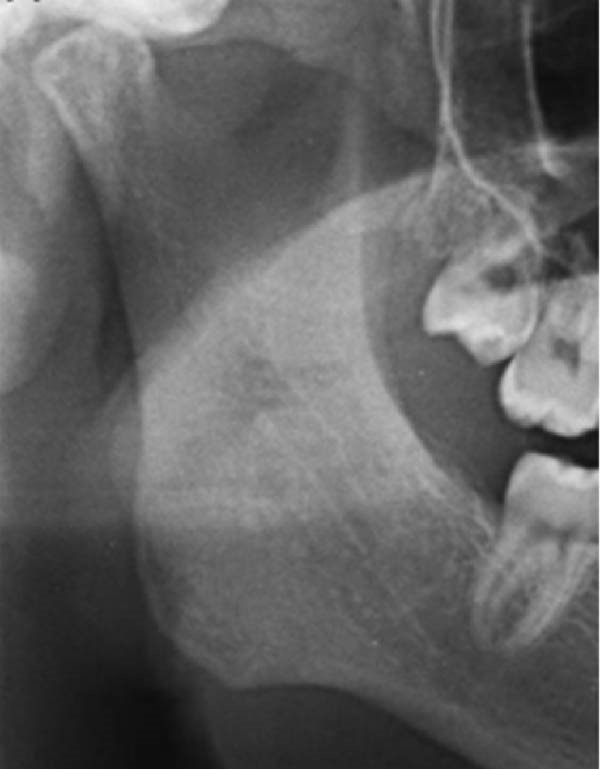
(A)

**Figure:**
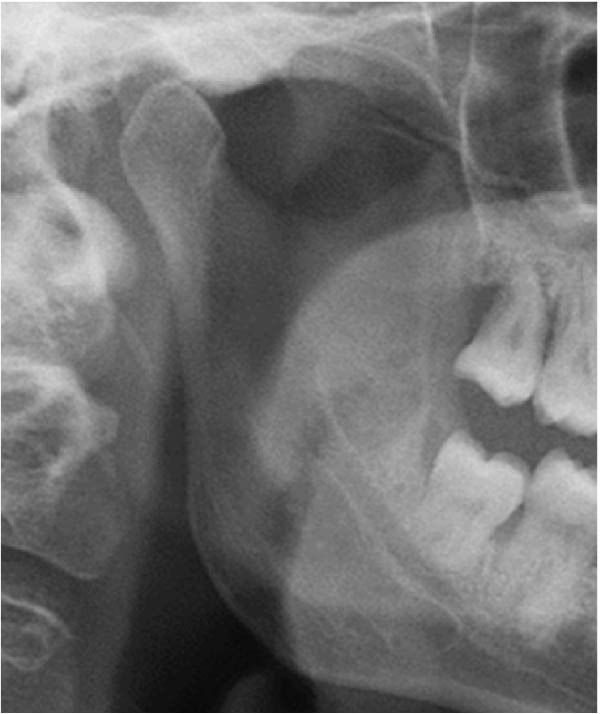
(B)

**Figure:**
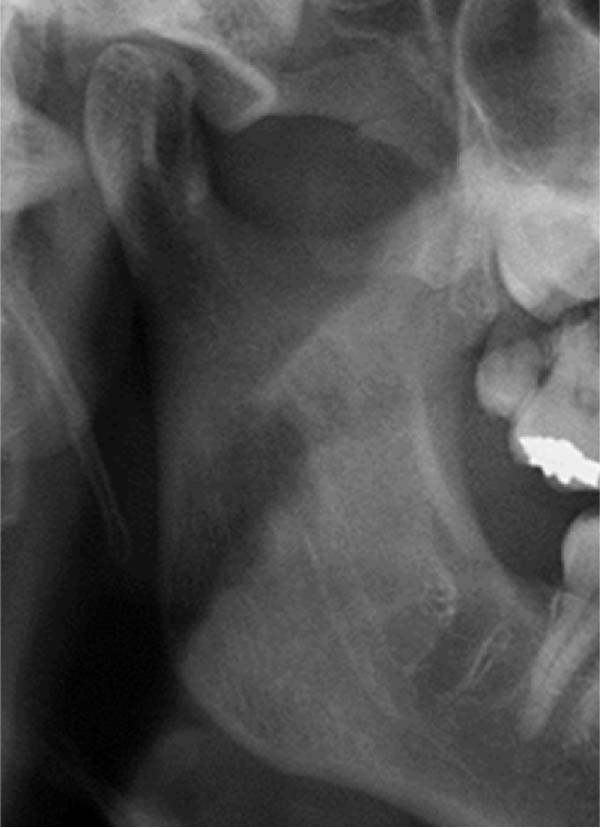
(C)

**Figure:**
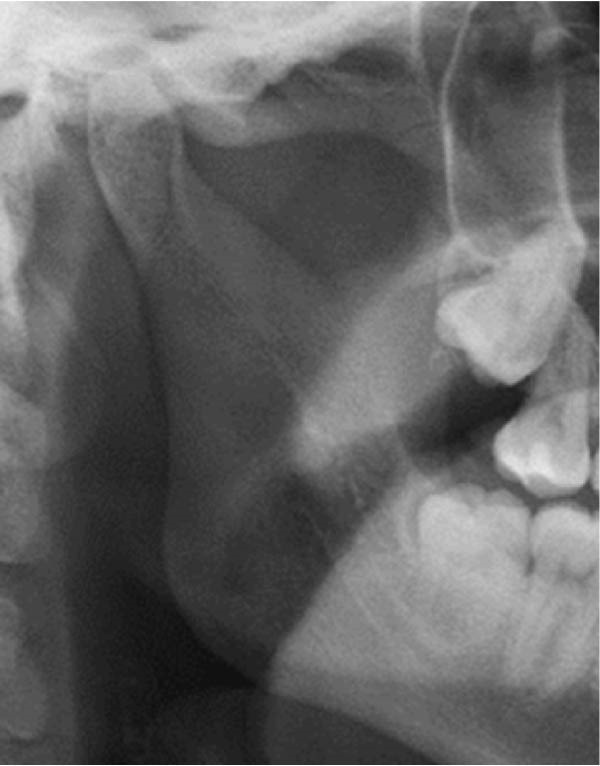
(D)

**Figure:**
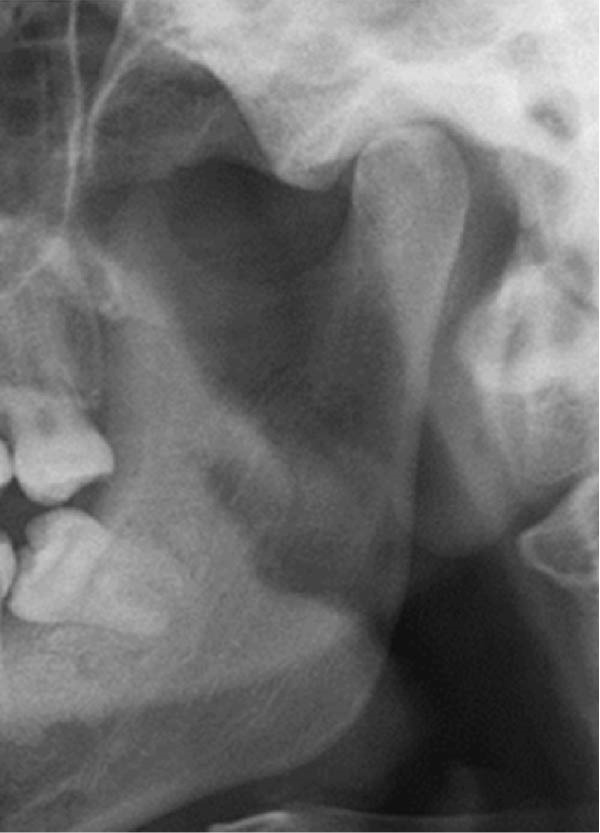
(E)

**Figure:**
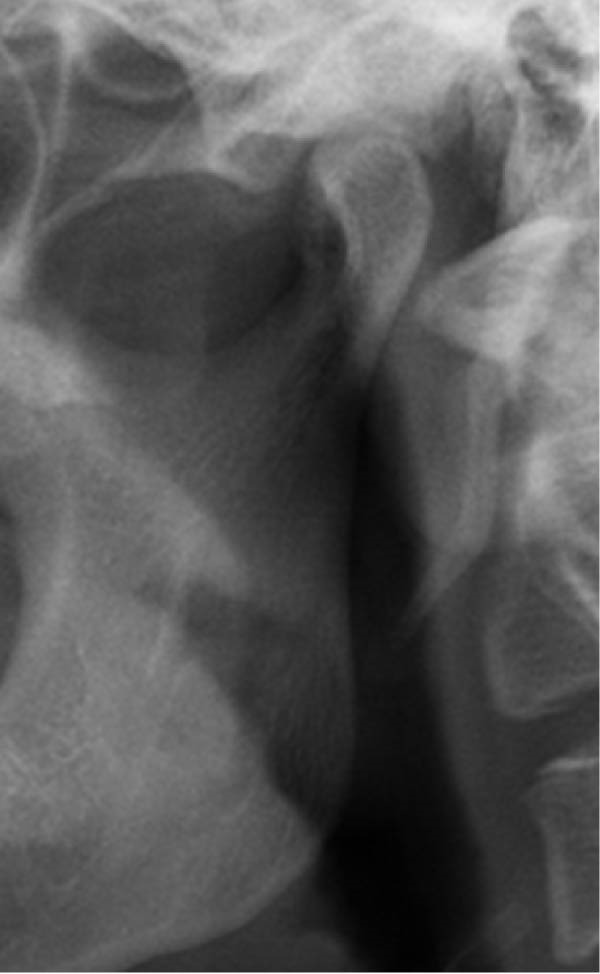
(F)

Figure 2Schematic illustration of different impacted mandibular third molar classifications based on Winter’s classification. (A) Distoangular, (B) horizontal, (C) mesioangular, (D) vertical,and (E) other types; buccolingual.

**Figure:**
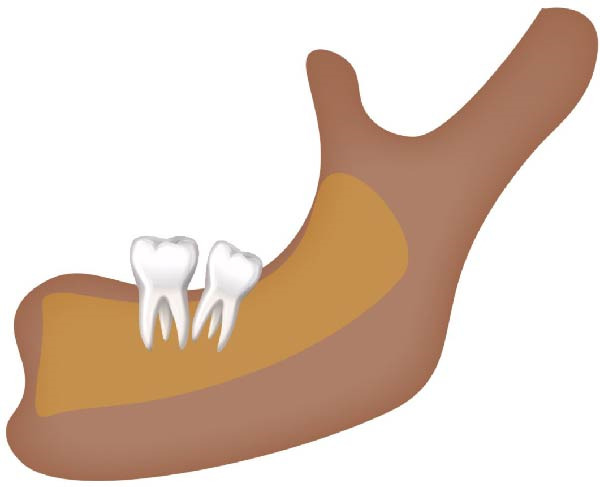
(A)

**Figure:**
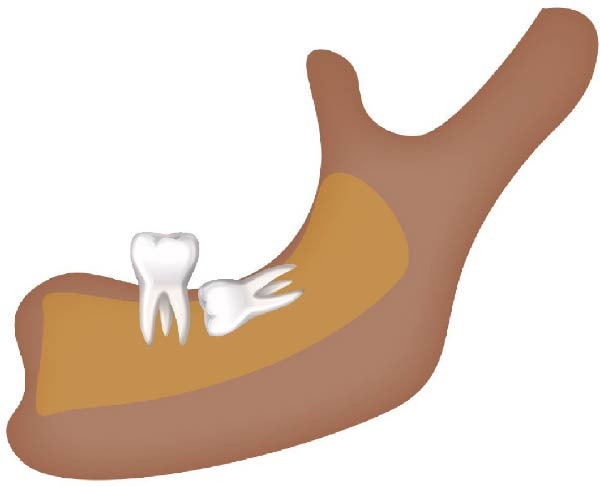
(B)

**Figure:**
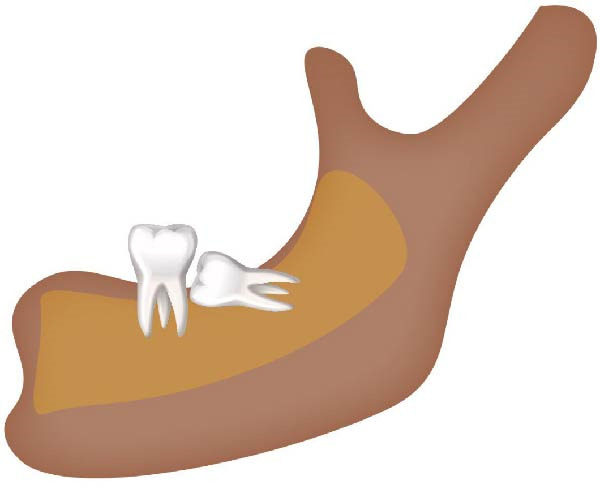
(C)

**Figure:**
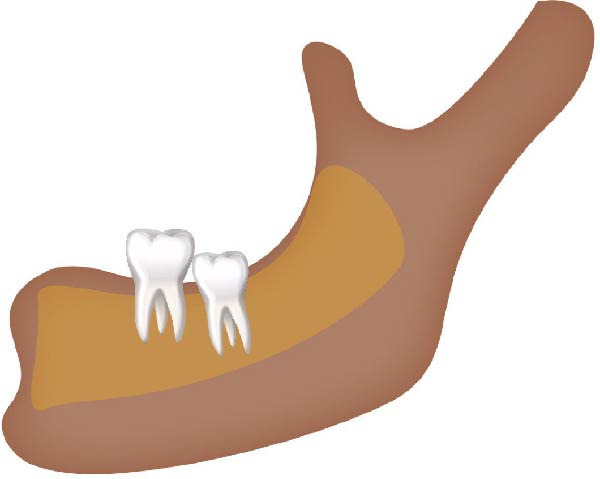
(D)

**Figure:**
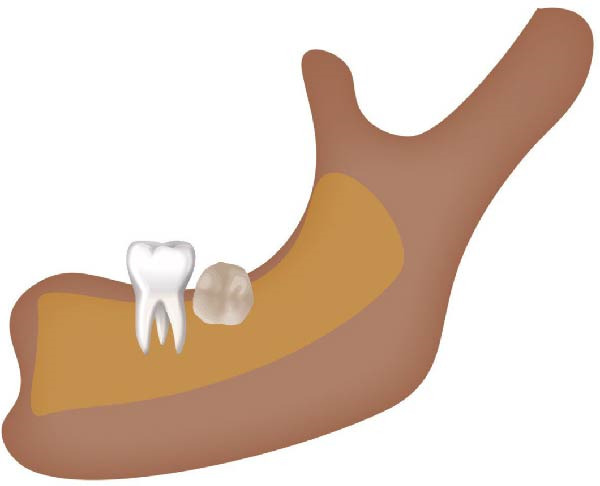
(E)

### 2.5. Criteria of Inclusion

Panoramic radiographs that had impacted the mandibular third molars of patients older than 18 years were included in the study. Images revealing any surgical deformity, trauma, or surgery of mandibular condyle were excluded from the study. Additionally, images of the patients younger than 18 years old and those of low quality and artifacts were excluded from the study.

### 2.6. Statistical Analysis

The descriptive statistics were reported as a frequency and percentage for the categorical variables with 95% confidence interval (CI), which is calculated using Wilson score method. Regarding continuous variables, the mean and standard deviation were reported. The independent *t*‐test was performed to compare the continuous measurements among males and females after the normality confirmation using Shapiro–Wilk test. Additionally, Chi‐square test as employed when the expected cell frequency is ≥5. Otherwise, the Fisher’s exact test is used instead. Regarding the calculation of the effect size, it is computed using Cramér’s *V* that is reported alongside *p*‐value to illustrate the association strength. Since we performed multiple bilateral comparisons (right vs left), we employed Holm–Bonferroni sequential correction to control error rate. To address the sparse cell, a sensitivity analysis was conducted, and the statistical significance was set at *α* = 0.05 (two‐tailed). SPSS Version 26.0 (IBM Corp., Armonk, NY, USA) was used to perform the statistical analysis.

## 3. Result

The present retrospective study revealed that out of 80 panoramic radiographs, 60% were of females and most of them (53.8%) were aged between 18 and 28 years (Table 1).

**Table 1:** Demographic distribution of the participants.

Variables	Age	Number (*n*)	Percentage (%)	95% CI
Age groups	18–28	43	53.8	42.3–64.9
	29–39	19	23.8	15.0–34.6
	40–50	4	5.0	1.4–12.3
	> 51	14	17.5	10.0–27.3
Gender	Female	48	60.0	48.4–70.7
	Male	32	40.0	29.3–51.6

*Note:* Data regarding age and gender according to number and percentage.

We also assessed the types of impactions associated with pathologies, condyle shape, and TMJ changes on the right and left sides of the mandible. We observed that on both sides, the vertical type of mandibular third molar impaction was the most common, and most of them either do not cause any pathology or a few lead to dental caries and bone loss. A maximum of 15% of cases showed sclerotic changes on both sides of TMJ. The most common shape of condyle was oval, followed by mixed. A statistically nonsignificant relation (*p* > 0.05) was observed between associated pathologies and condyle shape (Table 2). However, there was no statistically significant difference between the pathology associated with IMTM between right and left sides (*p* = 0.631; adjusted Holm–Bonferroni).

**Table 2:** Type of impaction and changes on the right and left sides.

Variables	Right side		Left side		Statistical analysis			
	Number (*n*)	Percentage (%)	Number (*n*)	Percentage (%)	95% (CI)	*X* ^2^	*p*‐Value	Cramér’s *V*
IMTM (winter’s classification)								
Absent	12	15.0	9	11.3	5.3–20.3	2.229^†^	0.816	0.12
Buccally tilted	1	1.3	0	0	—			
Distoangular	1	1.3	0	0	—			
Horizontal	12	15.0	13	16.3	9.1–26.0			
Mesioangular	22	27.5	24	30.0	20.4–41.3			
Vertical	32	40.0	34	42.5	31.5–54.0			
Associated pathologies								
Bone loss	40	50.0	36	45.0	33.8–56.6	3.448^†^	0.631	0.15
Bone loss and external resorption	1	1.3	1	1.3	0.0–6.8			
Dental caries	1	1.3	1	1.3	0.0–6.8			
Dental caries and bone loss	3	3.8	4	5.0	1.4–12.3			
No pathology detected	23	28.8	29	36.3	25.8–47.8			
Not applicable as tooth absence	12	15.0	9	11.3	5.3–20.3			
TMJ changes								
Erosion	5	6.3	5	6.3	2.1–14.0	1.126^†^	0.890	0.08
Flattening	9	11.3	6	7.5	2.8–15.6			
No change	53	66.3	55	68.8	57.3–78.9			
Osteophyte	1	1.3	2	2.5	0.3–8.7			
Sclerotic changes	12	15.0	12	15.0	8.2–24.7			
Condyle shape								
Bifid	6	7.5	7	8.8	3.6–17.2	3.715	0.591	0.15
Crooked finger	7	8.8	7	8.8	3.6–17.2			
Diamond	5	6.3	11	13.8	7.2–23.3			
Flattened	6	7.5	8	10.0	4.4–18.8			
Mixed	18	22.5	14	17.5	10.0–27.3			
Oval	38	47.5	33	41.3	30.3–52.8			
Total	80	100	80	100	—	—		

*Note:* All *p*‐values Holm–Bonferroni adjusted for multiple comparisons. Cramér’s *V*: effect size (0.1 = small, 0.3 = medium, 0.5 = large).

^†^Fisher’s exact test utilized in case the expected cell count is <5.

The prevalence of impaction was 86.88%, with the left side being the commonly involved side. Associated pathologies were observed with a prevalence rate of 54.37%, with most of them (56.25%) being observed on the right side (Table 3).

**Table 3:** Prevalence rate of the impaction and associated pathology.

Variable	Right side % (95% CI)	Left side % (95% CI)	Overall rate % (95% CI)
Prevalence impaction	85% (75.3–92.0)	88.75% (79.7–94.7)	86.88% (77.4–93.1)
Associated pathologies	56.25% (44.7–67.3)	52.5% (41.0–63.8)	54.37% (42.8–65.6)

Note: Data regarding the occurrence with respect to the corresponding side.

Association of types of IMTM was observed with different shapes of condyles on both the sides. On the right and left sides, oval‐shaped condyles were the most common. Vertical and mesioangular, followed by horizontally IMTMs were observed to be the most prevalent types of impactions, providing a statistically nonsignificant association (*p* > 0.05, Cramér’s *V* = 0.21–0.23) with condylar shape (Table 4).

**Table 4:** Occurrence of different shape of condyles with respect to the sides and type of impaction.

Right side	Bifid	Crooked finger	Diamond	Flattened	Mixed	Oval	*X* ^2^	Cramér’s *V* (95% CI)	*p*‐Value
Buccally tilted (*n* = 1)	0	0	0	0	0	1	12.45^†^	0.23 (0.05–0.38)	0.712
Distoangular (*n* = 1)	0	0	0	0	0	1			
Horizontal (*n* = 12)	1	3	1	0	1	6			
Mesioangular (*n* = 22)	0	1	0	4	6	11			
Vertical (*n* = 32)	3	3	4	1	8	13			

**Left side**									

Buccally tilted (*n* = 0)	0	0	0	0	0	0	14.28^†^	0.21 (0.08–0.41)	0.645
Distoangular (*n* = 0)	0	0	0	0	0	0			
Horizontal (*n* = 13)	0	1	1	0	3	8			
Mesioangular (*n* = 24)	2	1	4	3	6	8			
Vertical (*n* = 34)	4	4	5	3	4	14			

*Note: p*‐Values Holm–Bonferroni adjusted. Cramér’s *V*, effect size; 95% CI. No significant association detected between condylar shape and impaction type on either side.

^†^Fisher’s exact test was used because of the sparse cells.

## 4. Discussion

In craniofacial region, the mandibular bone occupies a very vulnerable and prominent position as the projected chin is a preferred target of trauma [17]. The mandible is like an archery bow, with the strongest part (symphysis) at its center and the weakest part (condyle) at its ends that makes it susceptible to fracture [18]. Due to its position in the posterior of the jaw, a relationship exists between increased chances of mandibular fracture and IMTM [19]. This is explained by the fact that IMTM can cause a decrease in the cross‐sectional area of bone, hence resulting in increased chances of mandibular fracture at the angle or condyle [18, 20]. Accordingly, assessing the impaction of mandibular molars is prudent. As mandibular third molar is the most frequently impacted tooth, its surgical management is also the most common practice in oral and maxillofacial surgery [21].

Several reasons contribute to the occurrence of the impaction such as inadequate space, abnormal positioning of the tooth, presence of supernumerary tooth, ankylosis of thedeciduous or permanent tooth, obstruction of the pathways of eruption by pathological conditions such as cysts or tumors, and external oblique line and buccinator muscleinfluences [22, 23]. It is also stated that the MSX1 and AXIN2 genes significantly mediate the occurrence of tooth impaction in coexistence of environmental factors and other modulating genetic expressions [24]. The present retrospective study revealed that out of 80 panoramic radiographs, 60% were females, and the majority (53.8%) were aged between 18 and 28. Like our study, Quek et al. [3] and Kumar et al. [5] observed female predominance, most commonly involving the age group 20–30. The higher prevalence rate in females is because of the varied growth patterns of males and females. When the third molar eruption begins, the growth of females usually stops, thus creating a rise in the prevalence rate of impaction [3, 5]. Among males, jaw growth continues during the eruption of the third molars, thus creating enough space for the eruption of the third molar. Similarly, Njokoma et al. [2] also observed that the most common age group with IMTM was 20–29 years. The high prevalence at this young age group is related to the eruption of the third molars, usually in late adolescence and early adulthood. In contrast to our study, Passi et al. [21] found male predominance in their study sample. We observed that the vertical type of IMTM was the most common on both sides. In contrast to our study, Hashemipour et al. [1] found that horizontal and mesioangular types of impaction were the most common. Passi et al. [21] found that mesioangular impaction was most common in the case of mandibular third molar teeth, whereas for maxillary arch, the most common type was vertical impaction. In accordance with our study, Bataineh et al. [24] and Almendros‐Marqués et al. [25] found that vertical impaction was the most common type of third molar impaction. The prevalence rate of impaction was 86.88%, with the left side being the commonly involved side. As reported in our study, Alfadil and Almajed [26] found a higher prevalence rate of 58.3%. The higher prevalence of impacted molars in our study may be because our study location is urban. Eating junk food and a soft diet causes a decrease in masticatory muscle activity on chewing, which causes hindrance in the growth and development of the jaw bones based on functional adaptation. We also assessed the types of impaction, associated pathologies, condyle shape, and TMJ changes on the right and left sides of the mandible. We observed that the most common associated pathology was dental caries and bone loss. A maximum of 15% of cases showed sclerotic changes on both sides of TMJ. Associated pathologies were observed with a prevalence rate of 54.37%, with most 56.25% on the right side. Like our study, Prajapati et al. [27] also observed dental caries followed by recurrent pericoronitis and periodontitis being the most common associated pathology with IMTM. This is because impaction can cause food impaction, thus leading to dental caries, periodontitis, and bone loss. Haddad et al. [28] found that distal caries was observed in 12.2% of mandibular third molar impaction cases. Our analysis used OPG as the radiographic technique of choice for evaluating the IMTMs. The radiation dose of OPG is also lower than that of four different periapical views and has a comprehensive diagnostic value in terms of revealing both maxilla and mandible in single image [29]. The disadvantage of OPG is that it has a lower diagnostic accuracy in detecting proximal caries. Because of this reason, there might be an underestimation of the incidence of dental caries in our study. In our study, the most common condyle shape was oval, followed by mixed. A statistically nonsignificant relation (*p* > 0.05) was observed between associated pathologies and condyle shape. An association of types of IMTM was observed with different shapes of condyles on both sides. On the right and left sides, oval‐shaped condyles were the most common. Shaikh et al. [15] and Varshan and Prathap [16] found that the most common condylar shape was oval, followed by bird beak, crooked finger, and diamond. In our study, vertical and mesioangular, followed by horizontally IMTMs, were observed to be the most prevalent types of impaction, showing a nonstatistically significant association (*p* > 0.05) with condylar shape. To date, we have not found any study observing the association of condyle shape with the type of mandibular impaction. Our study revealed that the most frequent impaction types are vertical, followed by mesioangular and horizontal. There was no statistically significant correlation with the condylar morphology. Futures studies using advanced imaging techniques such as cone beam computed tomography (CBCT) combined with TMJ assessment would provide an insight to further explore the association.

### 4.1. Study Limitations

The major limitations of this study are the small sample size, retrospective design, and convenience sampling. Thus, we advocate conducting prospective studies in the future with a larger sample size. We assessed panoramic radiographs for evaluation of impaction, but these are accompanied by various issues like missed pathologies, magnification, two‐dimensional view, and so forth. Thus, in the future, we advocate using CBCT with three‐dimensional views to assess better pathologies associated with impacted mandibular molars. Our study was based on hospital‐based records, which lacked randomization. The results of our study are affected by selection bias and could not be generalized to the whole population of Spain. More precise studies are required using a randomized sample representative of the population of Spain. As we assessed dental records, there are chances of retrieving incomplete data that might cause variation in the prevalence rate. In addition, further studies concerning parallel clinical assessment of TMJ disorders (TMDs) are essential. Although OPGs are not the optimal imaging modality for diagnosing TMJ condylar morphology with high validity, we attempted to minimize this limitation by including only images that are free of distortion and provided diagnostically acceptable quality and details. In addition, we perform the analysis within 2 months interval and calculating kappa, which provided substantial intra‐examiner agreement. Although our sample consisted of a relatively small number of OPGS and did not incorporate clinical TMJ symptoms, the study was intentionally designed as a radiographic analysis with the intention of integrating clinical manifestations and CBCT in the future work.

## 5. Conclusion

Our findings provide valuable baseline data for the type, pattern, and prevalence of mandibular third molar impaction in relation to age, gender, anomalies, and condyle shape among the Spanish population. The pattern of IMTM in the studied Spanish cohort showed the prevalence of vertical impaction, mainly on the left side of the mandible, with female predominance. With a high prevalence rate, we observed that commonly associated pathologies are dental caries and bone loss, revealing a nonsignificant relation in correlation with the type of impaction and condyle shape.

## Author Contributions

Hassan Ahmed Assiri wrote the entire manuscript. Albert Estrugo‐Devesa revised the manuscript. Sonia Egido‐Moreno performed language editing. Xavier Roselló Llabrés designed the methodology. Mohammad Shahul Hameed conducted the statistical analysis. Abdullah Alqarni revised the language editing. Jose López‐López supervised the whole process.

## Funding

No funds were received.

## Disclosure

A preprint has previously been published; Assiri et al. [30]. Jose López‐López approved the final version of the manuscript.

## Ethics Statement

The study was approved by the ethical committee of the University of Barcelona Dental Hospital (HOUB) under the number 32/2022. Consent to participants was waived by the ethical committee of the University of Barcelona Dental Hospital (HOUB) as we have only investigated anonymously the archived image of the patients without any intervention with the participants. All methods were carried out by relevant guidelines and regulations of the declaration of Helsinki.

## Consent

Patient consent was waived because we only reviewed the radiographic images of the patients anonymously, and there was no intervention or investigation on humans directly.

## Conflicts of Interest

The authors declare no conflicts of interest.

## Data Availability

Data are available upon request from the authors.
